# A Blow at the Germ Theory

**Published:** 1896-06

**Authors:** 


### A Blow at the Germ Theory.

The highest medical authorities are beginning to doubt the germ theory, and Dr. Laurie's latest discovery will make them more skeptical than before. Dr. Laurie claims that there is no parasite in the blood in malaria. If this fact be established it not only goes far to destroy the excellent reputation which the phagocytes enjoy for promenading the veins and devouring the malaria germ wherever found, but it is decidely unfavorable to the whole germ theory of disease. Malaria was supposed to be the one disease in which the action of the bacillus was said to be undoubted, and if it is disproved here it will be difficult to sustain it elsewhere.

